# Ethical and Clinical Aspects of Intensive Care Unit Admission in Patients with Hematological Malignancies: Guidelines of the Ethics Commission of the French Society of Hematology

**DOI:** 10.1155/2014/704318

**Published:** 2014-10-01

**Authors:** Sandra Malak, Jean-Jacques Sotto, Joël Ceccaldi, Philippe Colombat, Philippe Casassus, Dominique Jaulmes, Henri Rochant, Morgane Cheminant, Yvan Beaussant, Robert Zittoun, Dominique Bordessoule

**Affiliations:** ^1^Hematology Department of the René Huguenin Hospital, Institut Curie, 35 rue Dailly, 92210 Saint-Cloud, France; ^2^Ethics Commission of the French Society of Hematology, France; ^3^Hematology Department of the University of Grenoble, 38043 Grenoble, France; ^4^Hematology Department of the Robert Boulin Hospital, 33505 Libourne, France; ^5^Hematology Department of the University of Tours, 37044 Tours, France; ^6^Hematology Department of the University of Bobigny, 93000 Bobigny, France; ^7^Hematology Department of the University of Saint-Antoine, 75012 Paris, France; ^8^Hematology Department of the University of Créteil, 94010 Créteil, France; ^9^Hematology Department of the University of Necker, 75015 Paris, France; ^10^Hematology Department of the University of Besançon, 25030 Besançon, France; ^11^Hematology Department of the Hôtel-Dieu Hospital, 75001 Paris, France; ^12^Hematology Department of the University of Limoges, 87042 Limoges, France

## Abstract

Admission of patients with hematological malignancies to intensive care unit (ICU) raises recurrent ethical issues for both hematological and intensivist teams. The decision of transfer to ICU has major consequences for end of life care for patients and their relatives. It also impacts organizational human and economic aspects for the ICU and global health policy. In light of the recent advances in hematology and critical care medicine, a wide multidisciplinary debate has been conducted resulting in guidelines approved by consensus by both disciplines. The main aspects developed were (i) clarification of the clinical situations that could lead to a transfer to ICU taking into account the severity criteria of both hematological malignancy and clinical distress, (ii) understanding the process of decision-making in a context of regular interdisciplinary concertation involving the patient and his relatives, (iii) organization of a collegial concertation at the time of the initial decision of transfer to ICU and throughout and beyond the stay in ICU. The aim of this work is to propose suggestions to strengthen the collaboration between the different teams involved, to facilitate the daily decision-making process, and to allow improvement of clinical practice.

## 1. Current Situation

Therapeutic advances regarding hematological malignancies allow the care of an increased number and older patients and improve the chances of cure or prolonged remissions [1–7]. At the same time, aggressiveness of therapeutics is often associated with a high risk of clinical distress [8–10]. It has been reported that 7% of all new cases of hematological malignancies and up to 15% of acute myeloid leukemia may justify a transfer to ICU [11, 12]. The main reasons for ICU admission include acute respiratory failure, septic shock, acute kidney injury, and coma [13].

Advances in life-sustaining therapies improve the management of these patients, with increased knowledge of the chances of reversibility and of the risk factors of unfavorable outcomes [14–20], but mortality in this group of patients still ranges from 33 to 58% [14, 21–25]. Intensive care treatments may even allow, if necessary, continuing hematological treatments in the most appropriate environment [26–28].

The human and economic costs of critical care should prompt the reflection on the justification of each admission to ICU [29–32]. The information due to patients and relatives implies that they are informed and that their views are taken into account [33, 34]. The collaboration of the teams of hematology and intensive care should improve the necessary collegial decision-making process whose traceability is mandatory.

## 2. The Views of Hematologists

The clinical distress, particularly when it is not related to the expected evolution of the disease, is a situation where decision-making is of high importance for the hematologic team and where interpersonal difficulties can appear [35, 36].

The hematologists may have difficulties to estimate the severity of the sudden acute clinical condition and its chances of reversibility. Whereas some early warning scoring systems have been established to detect patients at risk of rapid deterioration and critical illness among general medical patients, they have been found inconsistently reliable in the hematological setting [37–39]. The risk could be to maintain the patient in hematology department instead of organizing an early transfer to ICU. On the other hand, increased requests for transfer to ICU for patients who will not benefit from this highly technological environment are not desirable [32, 40, 41].

To avoid these extreme situations, we propose to distinguish medical situations when hematologists should consider a transfer to intensive care units according to the expected evolution of the underlying condition, while knowing that there can be no rigid criteria to send patients to ICU and that this decision has to be individualized [13, 20].

It is usually admitted that characteristics of the underlying disease fail to predict short term survival [20, 42–49] but do influence longer term survival [50–52]. But recently, a large study demonstrated that remission status was correlated with in-hospital mortality [13].

The prognosis and the chances of reversibility depending on the nature and the extent of the organ failure are less predictable.

**Figure 1:**
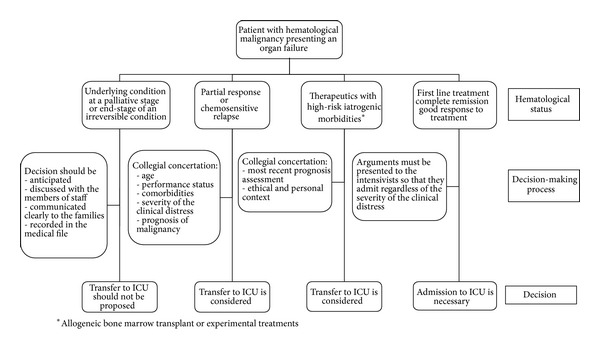
Decision model of ICU transfer of patients with hematological malignancies.

From the hematologists' point of view, we identified four admission situations (Figure 1).

(a) The hematologic underlying condition is at a palliative stage or (b) the patient suffers from an end-stage progressive condition unresponsive to any undertaken therapeutic measure, even if in remission of the hematologic disease (e.g., severe GVHD or progressive pulmonary failure). The acute illness is, then, the ultimate manifestation of an ineluctable deterioration. Usually, the transfer to ICU should not be proposed. It would be considered as an unreasonable and futile option. The decision of nontransfer should be anticipated, discussed with the members of staff, communicated clearly to the teams and the families, and recorded in the medical file.
The patient is (a) in first-line treatment and the therapeutic response cannot be assessed yet, (b) in complete remission or (c) showing a very good response to treatment; in those cases, the objectives of care are curative. The admission to ICU is necessary and the arguments must be presented to the intensivists so that they admit those patients regardless of the severity of the acute condition.
The patient is showing (a) a partial response, (b) a chemosensitive relapse, or (c) refractory to first-line treatments but with reasonable chances of efficacy with innovative further-line treatments. This represents the most difficult case in the decision-making process and justifies a thorough collegial concertation. The arguments to take into account include the patient himself (age, performance status, and comorbidities), the severity of the clinical distress, and the prognosis of the underlying malignancy.
The patient is involved in therapeutics with high-risk mortality and iatrogenic morbidities such as complications of allogeneic bone marrow transplant or experimental treatments. The decision-making process is usually complex and it should include the most recent prognosis assessment. The decision is not only technical and medical but also deals with the ethical and personal context that engages the responsibility of the hematologists prescribing procedures with potentially severe adverse consequences and for whom it may be difficult to assume disengagement.

## 3. The Views of Intensivist Physicians

In the past, the Society of Critical Care Medicine (SCCM) and the American Medical Association (AMA) [53, 54] clearly discouraged the admission of patients with oncological or hematological diseases to intensive care. This was especially true for patients requiring mechanical ventilation, with studies reporting more than 90% mortality in this population [55–58]. This led intensivists to have a negative image of patients with oncohematological conditions. These recommendations have been widely applied to adult patients for nearly ten years [59, 60].

Meanwhile considerable progress has improved the survival of these patients in ICU with an average mortality reduced to 40% including those requiring mechanical ventilation, dialysis, or shock therapy [15, 16, 22, 23, 43, 61]. As a result, the number of patients candidate for transfer to ICU increased considerably over the past years with the constant worry of doing the appropriate selection [62].

In the past, it has been reported that hematologic patients presented to the French intensivists for transfer to ICU had only 50% chances of being admitted [8]. More recently, 75% of patients considered for ICU admissions were finally admitted, but with 10% requiring more than one request before admission [13]. Interestingly, repeated requests were more frequent in patients admitted later, which may suggest a persistent reluctance to admit certain hematological patients. Besides a possible persistent negative image of the prognosis of hematological patients, this might be related to selection criteria that motivate refusal of admission to ICU.

These selection criteria differ between hematologists and intensivists. Hematologists consider in priority the underlying hematologic condition, the age of the patient, the performance status, and the availability of potentially life-prolonging treatments. Moreover, the concern about the infectious risk of neutropenic patients can lead to delaying the transfer, to maintain the patient in a protected environment. On the other hand, intensivists take into account the nature and the extent of multiple organ failures and favor early transfer to ICU so that patients can benefit from noninvasive diagnostic and therapeutic strategies before a potential deterioration of their clinical status [26, 63–65].

The difficulties arise from the fact that the information available at the admission to ICU is insufficient to discriminate the patients that will survive from those that will die [52, 66].

In order to maximize the chances of survival of the patients who may benefit from intensive care, different admission policies have been proposed. They are not necessarily exclusive of each other.

- Agreement on the level of care: also a single life-supporting intervention is associated with good survival; organ dysfunction appearing during the stay in ICU is associated with increased mortality [13, 25]. Consequently, in some cases, in particular when multiple variables predict a poor outcome, the intensivists and the hematologists may agree on the limitations of the level of care to deliver during the first days in ICU (e.g., noninvasive ventilation rather than endotracheal or nonactive treatment of a new organ failure such as dialysis in renal failure of a patient ventilated after a bone marrow transplant).
- ICU trial: a new strategy for admission of oncohematologic patients to intensive care entitled ICU trial as a politics of “*do everything that can be done*” [62], but for a limited period, has been elaborated instead of the well-known old strategy “*just say no*” [67, 68]. The ICU trial is an alternative to ICU refusal for hematologic patients that consists of unlimited ICU support during a limited period of time, where everything is done for at least 3 to 5 days [24, 63]. Considering the seriousness of such decisions and their potential impact on the patients' outcome, the ICU trial could be a solution that takes into account the ethical tension between utility and futility.
- Early transfer: this strategy favors early transfer to ICU so that patients can benefit from noninvasive diagnostic and therapeutic strategies before a potential deterioration of their clinical status. This approach is proactive rather than reactive and has been associated with improved outcomes [13, 61, 69].

## 4. Decision-Making Process

A structured process of decision making is critical to ensure consistency and the moral defensibility of these difficult decisions. The decision of transfer should arise from regular concertations between hematologists, intensivists, and their medical teams. The staff should be trained to be at a proactive interface with the patients and their relatives and to collect their consent for care and advance care planning [33, 34, 70].

The broad admission policies described earlier should lead to ICU admission for most patients within the scope of medical conditions 2, 3, and 4 described above that require life sustaining therapies because of at least one organ failure (other than hematologic failure) to define conditions of nontransfer (patients in palliative care, situation 1, or do-not-resuscitate order).

This project implies a multistep approach (Table 1).

- Early, when discussing the intensive hematological therapy, patients and their relatives must be informed of the risk of life-threatening evolutions, the consent for care must be obtained, and the patient's views about advance care planning should be reviewed.
- Intensivists must be consulted as soon as a clinical situation may require a transfer and they have to participate in the early detection of critical states to avoid taking decisions in emergency.
- The decision of transfer to ICU must arise from an interdisciplinary and collegial concertation between intensivists and hematologists, preferably in the daytime to avoid decisions taken in emergency or by a single physician as it could happen during the nighttime. The need to document patient preferences for resuscitation and end-of-life procedures is crucial before and at the admission to ICU.
- The decision of nontransfer falls within the general context of limitations of treatments in hematology. This involves mainly patients with poor life expectancy regardless of treatments and palliative care is then required to guarantee end-of-life quality [71–74]. The views of intensivists can be sought in critical situations even if hematologists do not consider formally the transfer to ICU.
- When a patient has been admitted in ICU, a concerted reevaluation must be programmed regularly. This assessment specifies the number of organ failures and redefines the objectives of care. Hematologists have to visit regularly their patients in ICU and should take part actively in the decision to maintain the patient in ICU.

Apart from patients who improved rapidly and are transferred back to hematology ward and those who died, the active collaboration between intensivists and hematologists mainly concerns the issue of extension of stay in intensive care that arises for other patients.

Different evolutions are possible [24, 44].

- The clinical state of the patient improves partially. Life support is pursued without limit, subject to regular concerted assessment.
- The clinical status deteriorates. The decision to withhold life-sustaining therapy should be considered. This is where the threshold between reasonable and nonreasonable stubbornness becomes an issue.
- Some of these patients will neither improve nor deteriorate with active life-sustaining therapies, with the same persistent organ failure as at admission. Those patients ultimately raise major issues for intensivists and hematologic teams but also for their family [75]. The medical decision has to be individual. Most often, life support is pursued with continuation of active hematological treatment if required, while indicating the relatives in an adequate way that any deterioration will not necessarily lead to therapeutic escalation. In all cases, it is essential to ensure that all means are sought to guarantee patient comfort and support for their families.

In case of limitation or withdrawal of active treatments, collegial concertation has to be maintained to initiate palliative care and patient accompaniment and to provide the appropriate support to the relatives [34, 76, 77]. At this stage, a transfer back to hematology ward could be organized for nonventilated patients. It is not there to abandon the patient but rather to facilitate the end-of-life in a quiet and comfortable environment, surrounded by the multidisciplinary team and the known caregivers, to allow his relatives to be free from the constraints of the ICU, and to facilitate psychological support. According to each situation, a transfer can be organized from hematology ward either towards a palliative care unit or back to the patient's home in collaboration with the family doctor.

## 5. Organization of the Concertation and Beyond

It is recommended to organize the concertation by integrating when possible the following procedures.

- On request of the hematologists, an intensivist can attend meetings in the department of hematology where high-risk procedures will be decided for the patients.
- It may be useful to appoint a referent intensivist that will be at a privileged interface with the department of hematology.
- Regular scheduled multidisciplinary meetings should be organized to evaluate the decisions and the collaboration, even retrospectively. The objective is to analyze clinical situations involving intensivists and hematologists, and their attitudes before the transfer to ICU, during the stay in critical care, after the release, and remotely beyond. These meetings would be opened to physicians and caregivers of both teams as well as to the palliative care specialists and psychologists. The aim is to identify areas of improvement.
- Multidisciplinary meetings of morbimortality conferences should be organized periodically.

All centers of hematology should establish a framework agreement according to their specificities with their referent ICU to define the rules of functioning, including staff training, as recommended by the Joint Accreditation Committee of the International Society for Cellular Therapy (ISCT) and the European Group for Blood and Marrow Transplantation (EBMT) [78]. Facilitating this collaborative work and the involvement of highly qualified personnel should be one of the priorities of the institutional management.

The organization of multidisciplinary concertation ahead of the admission decision as well as the development of information and communication should help in most cases to limit potential conflicts by anticipating them. Conflicts between physicians and patients or relatives may occur when there are decisions concerning the immediate future of the patient. Decisions to transfer or not to ICU may be perceived either as an aggressive treatment “*too much is done*” or on the contrary as abandonment and loss of chance* “not enough is done”* [75, 79, 80].

Immediate management of conflict and candid explanation to the patient or his relatives with a reasoned justification of the decision should help resolve the issues. If tension persists, it would be desirable to design a mediator to resolve the conflict. This mediator could be a member of the palliative care team, an ethicist, or a psychologist.

Investigations are still needed in the hematology units to monitor and evaluate the behavior and the factors influencing the primary decision of the hematologists to propose or not an admission to ICU, where selection criteria may vary according to the decision-making habits and the environment of each department. As a result, the only available research comes from ICU and includes only patients proposed to them. The objective would be to associate intensivists in the preliminary analyses of patients hospitalized in hematology, to organize a multidisciplinary dialogue, to anticipate the decisions, and thus to improve the identification of the patients that justify a transfer to ICU.

## 6. Conclusion

This work is the result of a collective reflection at the interface of two disciplines: hematology and intensive care and involves a common medical situation with decisions eminently difficult to manage in everyday life. Clarifying the medical conditions that may lead to a transfer to ICU, the relevant and consensual criteria of the decision-making process, and the concept of “ICU trial” represents an original aspect of this work.

The transfer of a patient has to respond to well-defined process on the basis of regular interdisciplinary collaboration before, during, and after the stay in intensive care. Decisions in this context have to comply with the principles of collegiality, with the involvement of the patient and his family and priority is given to anticipation approach. Traceability of decisions should also enable the individual and collective evaluation of these evolving professional practices. The evaluations of activities and regular meetings will allow maintaining communication between the professionals working in these departments and should lead to future collaborative research studies.

## Figures and Tables

**Table 1:** Multistep decision-making approach.

Before initiating any high-risk treatment	Possibility of a transfer to ICU should be discussed

As soon as a clinical distress appears	Intensivists must be consulted; they should participate in the early detection of critical states.

Transfer to ICU	Decision must arise from an interdisciplinary concertation between intensivists and hematologists. The need to document patient preferences is crucial.

Decision of nontransfer to ICU	Falls within the general context of limitations of treatments in hematology. Palliative care is required to guarantee end-of-life quality. The views of intensivists can be sought to help in symptom control.

3 to 5 days after admission to ICU	Concerted reevaluation must be programmed, especially in case of an ICU trial. Need to decide whether to maintain the same intensity of life-sustaining therapies or to consider withdrawal.

During stay in ICU	Hematologists have to visit regularly their patients in ICU and should take part actively in the decision to maintain the patient in ICU.

Regular scheduled multidisciplinary meetings	The objective is to discuss clinical situations involving intensivists and hematologists. It should be open to palliative care specialists and psychologists. The aim is to identify areas of improvement.

In case of limitation or withdrawal of active treatments	Collegial concertation has to be maintained to initiate palliative care and patient accompaniment and to provide the appropriate support to the relatives. At this stage, a transfer back to hematology can be discussed.
